# ACTOR: Adaptive Control of Transmission Power in RPL

**DOI:** 10.3390/s24072330

**Published:** 2024-04-06

**Authors:** Iliar Rabet, Hossein Fotouhi, Mário Alves, Maryam Vahabi, Mats Björkman

**Affiliations:** 1 School of Innovation, Design and Engineering, Mälardalen University, 721 23 Västerås, Sweden; hossein.fotouhi@mdu.se (H.F.);; 2 School of Engineering (ISEP/IPP), Politécnico do Porto, 4249-015 Porto, Portugal

**Keywords:** wireless sensor networks, Routing Protocol for Low-Power Lossy Networks (RPL), radio resource management, transmission power control, multi-armed bandit, reinforcement learning, Upper Confidence Bound (UCB), performance evaluation, simulation, testbed, IPv6, 6LoWPAN, IEEE 802.15.4

## Abstract

RPL—*Routing Protocol for Low-Power and Lossy Networks* (usually pronounced “ripple”)—is the *de facto* standard for IoT networks. However, it neglects to exploit IoT devices’ full capacity to optimize their transmission power, mainly because it is quite challenging to do so in parallel with the routing strategy, given the dynamic nature of wireless links and the typically constrained resources of IoT devices. Adapting the transmission power requires dynamically assessing many parameters, such as the probability of packet collisions, energy consumption, the number of hops, and interference. This paper introduces Adaptive Control of Transmission Power for RPL (ACTOR) for the dynamic optimization of transmission power. ACTOR aims to improve throughput in dense networks by passively exploring different transmission power levels. The classic solutions of bandit theory, including the *Upper Confidence Bound* (UCB) and *Discounted UCB*, accelerate the convergence of the exploration and guarantee its optimality. ACTOR is also enhanced via mechanisms to blacklist undesirable transmission power levels and stabilize the topology of parent–child negotiations. The results of the experiments conducted on our 40-node, 12-node testbed demonstrate that ACTOR achieves a higher packet delivery ratio by almost 20%, reduces the transmission power of nodes by up to 10 dBm, and maintains a stable topology with significantly fewer parent switches compared to the standard RPL and the selected benchmarks. These findings are consistent with simulations conducted across 7 different scenarios, where improvements in end-to-end delay, packet delivery, and energy consumption were observed by up to 50%.

## 1. Introduction

The increasing adoption of Low-Power and Lossy Networks (LLNs) as a leading class of Internet-of-Things (IoT) communication technologies has led to a high density of wireless nodes in the already congested unlicensed industrial, scientific, and medical (ISM) bands. LLNs are typically composed of battery-operated and resource-constrained devices connected via a mesh network over unreliable links. For LLNs, the *Internet Engineering Task Force* (IETF) has proposed a protocol stack consisting of the Routing Protocol for LLNs (RPL) [1] and *IPv6 over Low-Power Wireless Personal Area Networks (6LoWPAN)* [2]. RPL targets scaling to thousands of IP-connected devices, to fulfill the vision of IoT. Modern IoT applications not only require the scalability and lightweight operation that RPL provides but may also impose a heavy traffic load and dense deployments. e.g., habitat monitoring [3], underground mining [4], and smart grids [5].

However, this protocol stack shows degraded performance when subjected to high traffic or a dense topology [6]. In such cases, RPL solicits information from other possible parents and switches to another parent. Parent switching might not always enhance the network performance, particularly in densely deployed networks, since the potential parents may also perform poorly.

One reason is that in RPL networks, the predominant choice is to set the transmission power to the maximum for all the nodes to simplify the operation of the protocol. Alternatively, the wireless nodes can adjust their transmission power to increase spatial reuse in the network consequently improving the quality of service under dense and heavy traffic scenarios. First, it may increase channel contention and reduce spatial reuse in the channel. Consequently, nodes waste their limited energy due to (i) unnecessary large values of transmission power and (ii) the re-transmission of packets due to collision with other nodes. On the other hand, radically reducing the transmission power may compromise the network connectivity since receivers may experience a lower Signal-to-Noise Ratio (SNR) [7].

Commercially available off-the-shelf (COTS) radios provide metrics such as the *Receiver Signal Strength Indicator* (RSSI) and *Link Quality Indicator* (LQI). These metrics provide an instantaneous metric to decide on the transmission power but are volatile in nature and not very accurate for the long-term estimation of wireless link quality [8,9]. That is why accurate methods require nodes to probe different transmission power settings continuously, which implies severe overhead to the network. Moreover, adding extra control traffic escalates congestion, which is the underlying cause of packet loss and delay. By passively measuring the delivery rate of various settings, node can infer the link quality without requiring additional control traffic. Passive probing can be costly if it is not performed properly, as nodes may sacrifice some data packets by reducing the transmission power for the sake of exploring the opportunity. Some existing works probe different transmission powers using simple algorithms without providing a theoretical background on convergence to the optimal setting. For instance, window-based protocols [10] assume they have found the best transmission power if they successfully transmit a certain number of packets. Our preliminary study shows that, even in a simple two-node scenario, this assumption is unrealistic. In such settings, these algorithms might not select the optimal transmission power or converge very slowly.

An intelligent exploration strategy is required to ensure the probing of different settings is performed faster and is guaranteed to converge. We resorted to the exploration–exploitation dilemma in the *Multi-Armed Bandit* framework and UCB algorithm to optimize the probing of the transmission power settings. *Multi-Armed Bandits* are a class of reinforcement algorithms in which a single-state agent has no prior knowledge of the rewards. In this framework, each node simultaneously learns and optimizes its rewards.

We present *Adaptive Control of Transmission Power for RPL* (ACTOR), which includes a set of methods that extend RPL to adapt transmission power settings based on bandit theory. ACTOR aims to increase the throughput while minimizing the transmission power in dense and high-traffic LLNs. This also has the potential to preserve energy in the batteries through spatial reuse and lower internal interference.

ACTOR takes advantage of the routing information in the transmission power control. The reliable communication of nodes in a multi-hop network hinges on the parents to relay the traffic. When a parent node reduces its transmission power, it is not supposed to jeopardize communications with its children. In ACTOR, children inform their preferred parent of their expected transmission power settings so the parents do not abandon their children. To further accelerate the convergence of the learning process, ACTOR also employs a blacklisting mechanism to filter out transmission power settings that are too unreliable. Importantly, ACTOR is backward-compatible with the RPL protocol and easy to implement in COTS devices with minimal overhead.

**Figure 1:**
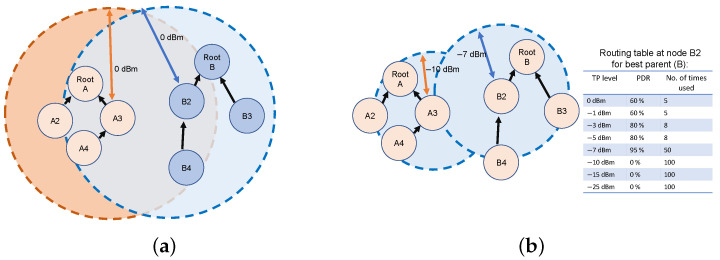
ACTOR confines the channel contention among nodes in a multi-cluster topology. (**a**) The clusters interfere with each other when using the maximum transmission power. (**b**) The clusters have minimal interference with each other when using ACTOR.

Transmission power control is advantageous in many scenarios. In a multiple-cluster network, ACTOR is beneficial since it allows the clusters to limit their destructive interference to a smaller region. Figure 1 illustrates a multi-cluster topology in which the two clusters do not interfere with each other when using ACTOR (Figure 1b). When running RPL, the transmission ranges of nodes A3 and B2 collide with a lot of nodes in the neighboring cluster (Figure 1a). The figure also shows a sample of the table that ACTOR maintains for storing the action values and the number of times they have been explored. By using ACTOR, nodes can improve their throughput in dense or high-throughput scenarios while minimizing their energy consumption. This is achieved through optimal transmission power control, fast convergence, and keeping the topology stable.

The recent efforts in the domain of deterministic networking in the IoT have led to the standardization of the *Time Slotted Channel Hopping* (TSCH) and IPv6 over TSCH (6TiSCH) framework [11]. The benefits of increasing spatial reuse in the network are not limited to tackling the hidden terminal problem in a CSMA network. Avoiding excessive transmission power settings reduces the interference incurred to the neighboring nodes. Hence, even with the proliferation of TSCH mode in the MAC layer, the transmission power control reduces the constraints of the scheduler, allowing higher throughput.

In summary, this paper outlines the following contributions:

- We experimentally analyze packet delivery ratio distribution with different transmission powers and distances for two nodes communicating with RPL, showing the need for intelligent transmission power control (Section 4).
- We formulate the problem of transmission power control in RPL using the *multi-armed bandit* paradigm and propose the ACTOR mechanism with two alternative algorithms—UCB and Discounted UCB (Section 5).
- We implement and integrate the ACTOR mechanism in RPL in a way that is backward-compatible with the standard protocol using Contiki-NG, and we publish the code as open-source (https://github.com/iliar-rabet/ACTOR, accessed on 3 April 2024 ) (Section 5).
- We evaluate and compare ACTOR performance against the default RPL and two benchmarks, both through simulations and a real testbed, showing improved reliability and energy efficiency (Section 6).

The remainder of the paper is structured as follows: Section 2 reviews some background regarding bandit theory and the standard RPL protocol. Section 3 addresses the related work. In Section 4, we analyze the upper bounds of the distribution of the PDR using different transmission power settings. Section 5 addresses the proposed method, which is then evaluated in Section 6. Finally, we conclude the paper in Section 7.

## 2. Background

ACTOR extends the standard RPL to optimize the TP using a specific reinforcement learning algorithm named UCB. So, in this section, we first review the standard RPL and its core mechanisms. Next, we present the reasoning behind choosing the UCB algorithm among all reinforcement learning schemes.

### 2.1. Background on RPL

All transmissions (upward, downward, and point-to-point) in RPL happen within a set of links referred to as a *Destination-Oriented Directed Acyclic Graph* (DODAG). RPL nodes construct a collection of DODAGs, each rooted at a border router. For each DODAG, the root node starts the process by sending a *Destination Information Object* (DIO) indicating its presence. Upon receiving a DIO, nodes can start sending data to the root and set a Trickle timer to further advertise the network by sending more DIOs. Nodes also send *Destination Advertisement Object* (DAO) packets to the root node to enable downward traffic to be routed.

Numerous RPL extensions, both centralized [12] and distributed [13], have been proposed in the research community to address various challenges such as network density, mobility, and interference. While centralized approaches offer certain advantages, such as centralized control, they often introduce additional control traffic, which can be undesirable in dense networks. Therefore, to mitigate these concerns, ACTOR proposes enhancements to the standard in a distributed manner.

RPL was not initially tuned to cope with channel contention. In the event of packet loss due to high traffic, RPL tries to initiate local or global repairs by sending more DIOs, thereby further congesting the medium. Local repair refers to the scenario in which a node starts searching for a parent with no implication on the global routing state. This is triggered when the node does not have any active link toward the root node. A global repair, however, can only be initiated via the root node, and it rebuilds the whole routing graph on all the nodes from scratch. It has been shown that, under a heavy traffic load, RPL shows unstable topology maintenance, unfairness, and high routing overhead. Also, handling the hidden terminal problem is flawed since nodes do not use their capacity to adapt their transmission power.

RPL nodes advertise the quality of their upward connection using an *Objective Function* (OF). The *Objective Function 0* (OF0) and *Minimum Rank with Hysteresis Objective Function* (MRHOF) are well-known, famous OFs mostly used with different routing metrics, including the expected transmission count (ETX), RSSI, and nodes’ remaining energy. Among the metrics, ETX is the most commonly used, as it captures both the long-term link reliability and energy consumption. The calculation of the routing metric assumes constant transmission power in the standard RPL.

### 2.2. Bandit Theory

The classic *Multi-Armed Bandit* problem can be formulated as follows. The agent repeatedly chooses from a set of actions (arms) with rewards that are initially unknown and observes the associated reward. In the next iterations, the agent uses the knowledge it has gained during the previous iterations. The goal of the agents is to minimize a regret function, which is mathematically formulated as the difference between actual accumulated rewards (with unknown statistics) and the optimal reward [14]. In parallel with optimizing the accumulated reward, the agent aims to learn about the environment. The optimal policy consists of always choosing the action with the maximum expectation of reward. Intuitively, the faster an agent identifies the best action, the less regret it is likely to have.

Bounding the regret can be realized by balancing the tradeoff between exploration and exploitation. Exploration can be defined by examining arms that have not been used before. If the agents overspend their resources by excessively exploring actions, they lose long-term rewards and increase their regret. Burnetas et al. [15] proved a lower bound for the number of times that each arm needs to be pulled to learn about the environment. This translates into an instance-independent lower bound of the regret of the agents. This lower bound is usually used as a measure to assess the robustness of an exploration policy.

Unlike the classic ε-greedy, modern exploration policies such as the *Upper Confidence Bound* (UCB) bring about fast convergence and optimal results. The intuition behind the UCB algorithm is based on a principle called optimism in the face of uncertainty. Agents employing this algorithm always take the action that is as large as plausibly possible. Auer et al. [16] proved that UCB achieves sublinear regret in time, outperforming the linear regret associated with ε-greedy and matching the lower bound achieved by Burnetas [15].

Depending on how the problem is formulated, bandit theory proposes different solutions and methods, including non-stationary bandits, adversarial bandits, and contextual bandits. Non-stationary bandits can be applied when the distribution governing rewards is dynamic. Secondly, for the adversarial version, the distributions not only are non-stationary, but also, an adversary is trying to work against the agent by reducing the rewards. The algorithms that are designed for these classes of problems are Discounted UCB and EXP3, respectively. For the contextual bandits, the agent associates one model to each context, and when it detects the context, it uses its associated model to choose the action.

### 2.3. Upper Confidence Bound Algorithm

In this subsection, we calculate the upper bounds for the family of UCB algorithms, which is a delicate matter. These bounds are typically derived using a statistical theory called concentration of measure. In this theory, exponential tail inequalities play a central role in analyzing the tail of the distributions and, thus, finding tight upper bounds. Choosing among the variants of UCB revolves around the defined bounds and their tightness [17]. The basic UCB algorithm relies on the concept of subgaussian random variables. For a σ-subgaussian random variable, the tail decay is approximately as fast as that of a Gaussian with the same variance.

**Definition** **1.** *A random variable, X, is said to be σ−subgaussian if it holds that*

(1) $$E\left[\exp\left(\lambda X\right)\right]\leq \exp\left(\lambda^{2}\sigma^{2}/2\right)$$

In this paper, the delivery of the packets is the reward, and different transmission powers are the arms. Given that PDR is always bounded to [*0*, *1*], one possible upper bound can be derived using the following bound:

**Lemma** **1.** *If a random variable, X, has a mean zero and X∈[a,b], then X is (b−a)/2-subgaussian.*

Another slightly tighter bound can be obtained using *Hoeffding’s inequality*, which states that X is ${\left(b-a\right)}^{2}/4$-subgaussian for a zero-mean [a,b]-bounded random variable, X. (For proof, see the Appendix A.)

When it is known that the underlying distribution of rewards is σ−subgaussian, the relation between the probability of violating and the bound is as follows [17]:

(2) $$P[X\left(t\right)\geq {\bar{X}}_{t}\left(a\right)+\sqrt{\frac{2\sigma^{2}\log\left(1/\delta \right)}{N_{t}\left(a\right)}}]<\delta$$

Assuming some values for delta (usually $\delta =1/t^{2}$), we can calculate the UCB:

(3) $$UCB_{i}\left(t,\delta \right)=\left\{\begin{array}{cc} \infty & \mathrm{if}\;N_{i}\left(t-1\right)=0\\ {\bar{X}}_{t}\left(a\right)+\sqrt{\frac{2\sigma^{2}\log\left(1/\delta \right)}{N_{t}\left(a\right)}} & \mathrm{otherwise} \end{array}\right.$$

Building upon this upper bound, the UCB algorithm runs a simple loop which for iteration (*t*) consists of the following operations:

1.   Choose the Action(t) = argmaxUCB(t−1)
2.   Observe $X_{t}$ and update the bounds

Assume $\Delta_{i}$ is the suboptimality gap or the immediate regret for action *i*, and $N_{i}\left(t\right)$ is the number of times arm *a* has been pulled before time *t*. Then, regret, $R_{n}$, can be formulated as follows:

(4) $$R_{n}=\sum_{a\in A}\Delta_{i}E\left[N_{a}\left(t\right)\right]$$

The theorem below presents an analysis of the regret when using the UCB algorithm. In the rest of this paper, by UCB algorithm, we mean UCB(δ). For a regret analysis of the discounted version of UCB, please refer to [18].

**Theorem** **1.** *Consider the UCB algorithm in a stochastic 1-subgaussian k-armed bandit problem. For any time horizon, t, when accepting an error probability of δ, then the regret, $R_{n}$ (defined in Equation (4)), is bounded by the following:*

$$R_{n}\leq 3\sum_{i=1}^{k}\Delta_{i}+\sum_{i:\Delta_{i}>0}\frac{16\log n}{\Delta_{i}}$$

This theorem ensures that the regret growth of UCB is sub-linear relative to time.

## 3. Related Works

This section reviews the previous works in the field. First, we address the efforts to jointly optimize the transmission power and routing. Then, we focus on research works that employ reinforcement learning methods for transmission power control, and at last, the two selected benchmarks are reviewed.

### 3.1. Transmission Power Control and Routing

A significant body of research investigates transmission power control. Santi [19] provides a survey on a wealth of research on transmission power control. The majority of these research works focus on theoretical aspects, including maintaining graph properties such as k-connectivity. A major gap that authors notice in the literature is that the theoretical transmission power control algorithms have not been efficiently integrated with the standard protocols (including RPL).

Lin et al. [20] classify the literature on transmission power control into approaches that select the transmission power for (i) the whole network, (ii) each node, and (iii) each pair of nodes. The authors also introduce *Adaptive Transmission Power Control* (ATPC), and at run time, the transmission power is set for each pair adaptively. Pairs build a linear model to map the transmission power to the RSSI at the destination. To this end, nodes gather samples by sending a set of beacons at different transmission power settings and adopt a least-squares approximation to find the two coefficients for the linear model (slope and bias). Based on the empirical results, the authors analyzed how the linear model changes over time. The slope is shown to be almost constant for the same physical environment, but the bias changes more significantly over time. So, after initializing the model according to the approximation, pairs update the bias to cope with the link dynamics over time. The fact that ATPC nodes piggyback RSSI readings (rather than ETX or PDR) is an important design choice, as it may be impossible to retrieve all the RSSI readings due to interference. On the other hand, ACTOR aims at reducing the size of the neighbor table by only evaluating the links for possible parents and reasonable transmission power settings.

While ATPC and many of its counterparts lack integration with RPL, the most prominent works that extend RPL to adapt transmission power control are PC-RPL [10] and XRPL [21]. We studied these two proposals in more detail as the selected benchmarks. The remaining works in the literature that focus on this research problem are as follows.

Barcelo et al. [22] extend RPL to jointly perform routing, channel selection, and transmission power control. The proposed method consists of two strategies, namely the *Maximum Probability Delivery Ratio* (MaxPDR) and the *Minimum Aggregated Power* (MinAP). For both strategies, DIO packets are sent in batches (rather than a single packet) using all the available power levels. Based on the reception ratio of the DIO batch, nodes select their transmission power before making any routing decision. MinAP prioritizes energy consumption over the reliability of the network. Under MaxPDR, however, first, the node with the highest end-to-end PDR gets selected as the parent. Then, the transmission power is configured to the lowest level that yields sufficient PDR (for example, 80% of the maxPDR). The drawback of both MaxPDR and MinAP is the increasing number of DIO packets that introduce a tangible communication overhead.

TCOR [23] combines an opportunistic routing approach with transmission power control to reduce energy expenditure while maintaining link reliability. In this theme, the transmitter keeps retransmitting its packets until one of the forwarders acknowledges the packet. To select the appropriate forwarders set and their associated transmission power, nodes estimate the probability of packet delivery based on a shadowing propagation model.

Adaptive Robust Topology control (ART) [8] defines a sliding window and two thresholds. If the ratio of correctly acknowledged packets during the sliding window is high, it tries to reduce the transmission power in a “trial” state. If the reception ratio falls, ART may increase or decrease the transmission power based on history. Ko et al. [24] take a similar approach by actively probing the transmission power settings. In the active probing mechanism, if a certain number of packets is transmitted successfully, the transmission power is decreased by one unit. If any of the packets are not acknowledged, the nodes increase the transmission power. These two proposals are based on the same assumption that a link is reliable if it is able to successfully transmit a certain number of consecutive packets. Our preliminary study shows situations in which this definition of a good link is not useful. Neither of these two proposals is integrated with RPL.

Miguel et al. [25] adjust the transmission power to keep the size of the parent table as close as possible to a predefined threshold. Their proposed DODAG-oriented method was designed to be executed offline. The authors also mention the problem of the initialization of ETX, which can be optimistic/pessimistic.

### 3.2. Applications of Reinforcement Learning in Link Quality Estimation

Estimating radio link quality plays an essential role in both routing and topology control. A comprehensive study of the wireless link behavior in a low-power setting is conducted in [26]. The lossy and time-varying nature of the radio links requires frequent evaluation of the link quality towards each neighbor. Hence, among different classes of machine learning algorithms, Reinforcement Learning emerges as the most suitable mathematical framework for real-time and adaptable resource optimization. Li et al. [14] conduct a survey that delves into applying bandit theory, a pivotal problem domain within Reinforcement Learning, specifically in the domain of scheduling wireless links.

QL-TPC [27] models the transmission power control problem as a Markov Decision Problem (MDP). The authors apply Q-learning, which is a Temporal Difference method, to find the optimal transmission power. The states are defined by the number of retransmissions and *Clear Channel Assessments* (CCA) attempts, and rewards are determined when receiving acknowledgments. QL-TPC employs ε-greedy as the exploration strategy, which introduces sub-optimal exploration.

A basic solution for the exploration–exploitation dilemma is the famous ε-greedy algorithm, which has a rich history of being used in wireless systems [14]. Nodes choose a constant value for ε, and in each slot, either select the highest rewarded arm in a greedy approach using probability 1-ε or randomly select among other arms to explore using probability. The selection of the arms in the exploration phase is purely random.

ε-greedy has two major shortcomings that more advanced solutions try to overcome. First, the exploration continues worthlessly even when it is obvious which actions are better rewarded. Second, the agent naively takes random actions in the exploration phase and discards all the retrieved information.

Maghsudi et al. [28] model the problem of channel and transmission power selection using adversarial bandits. In these types of bandit problems, the probability distribution of the rewards cannot be attributed to any static distribution since an adversary is opposing the agent. Nodes that incur interference with each other when selecting transmission power are modeled as adversaries. If all agents (nodes) take a strategy with vanishing regret, the joint distribution converges to an equilibrium. Hence, they employ two such strategies, namely the exponential-based weighted average and Follow The Perturbed Leader (FTPL). The main idea of the former is assigning a probability for the selection of actions that is proportional to the accumulated regret. In the latter strategy, agents add a random perturbation to the regrets and then select the action with minimum accumulated regret.

Aboubakar et al. [29] employ a multi-layer perceptron (MLP) model to estimate the optimal transmission power in RPL networks with various topologies. This necessitates model training through an offline approach and a labeled dataset.

Sohail et al. [30] formulate the problem of selecting an efficient cluster head as an evolutionary game. Each node is modeled as a self-interest agent that continuously adapts its strategy to maximize energy efficiency. This approach aims to strike a balance between the remaining energy, hop level, density, and degree of connectivity. This proposal is not integrated with the RPL protocol stack.

In the context of Wireless Body Area Networks, the anatomical constraints of body movements, and the orientations measured via motion sensors can be exploited in favor of topology control. Tuatara [31] takes such an approach and employs learning automata as a learning technique to optimize the transmission power.

In the context of cellular IoT and massive MIMO networks, the problem can be approached using methods such as Channel Inversion, which adjusts the transmitted power inversely proportional to the channel gain. An alternative approach is the Max Min power control scheme, for which the main idea is to find an optimal power allocation strategy that maximizes the minimum SNR among all users [32]. However, transmission power control is fundamentally more challenging in a multihop mesh network with the presence of a routing protocol.

### 3.3. The Benchmarks

Among numerous proposals in the literature, the most relevant benchmarks are those that are integrated with RPL. As a benchmark, ACTOR has been compared to two recent works: TPP and XRPL.

#### 3.3.1. Transmission Power Probing (TPP)

Our first benchmark implements the window-based technique which is common in the literature [8,10,24]. Nodes select and keep a transmission power and during a window certain number of packets are transmitted using the selected transmission power. This *window-based* mechanism is dubbed as *transmission power probing* (TPP) throughout the paper. By selecting TPP as benchmark we can check if ACTOR’s probing is more efficient. Our implementation of TPP closely follows a recent work titled PC-RPL [10]. TPP was implemented by the authors since similar works were either not integrated with RPL or their implementation was not available. To the best of our knowledge, three previous works have employed variations of the window-based probing approach: PC-RPL [10] is a recent study that jointly performs transmission power control and routing. In PC-RPL, DIO packets are sent with the maximum transmission power, but for the data packets, the transmission power is decided at run-time using a window-based adaptive mechanism. When joining the network, the measured RSSI is used to initialize the transmission power. Then, at run-time, if the node succeeds in transmitting *M* consecutive packets, it will try reducing the transmission power by 1 level. Upon packet loss, PC-RPL increases the transmission power by 2 levels and doubles *M* to make future decisions more conservative. Despite the previous works, PC-RPL consolidates the transmission power control with a load-balancing mechanism in which parent nodes can inform their children to switch to another parent. The load balancing is advantageous in combination with transmission power control since the parent node will not be committed to serving the children. Unfortunately, the implementation was not available to reproduce the results.

**Figure 2:**
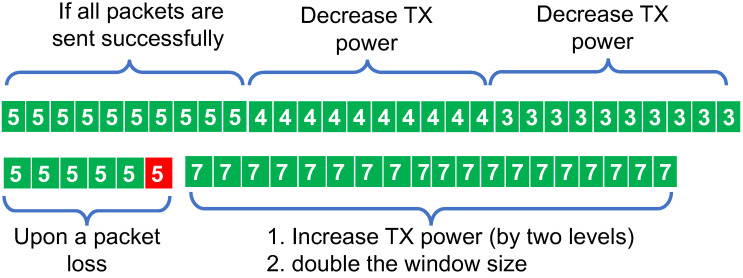
Transmission Power Control in TPP.

All three of the mentioned works define “reliable” links as those that deliver a certain number of successful packets consecutively. In a scenario in which all of the transmission power settings are less than ideal, this definition is not useful. For example, consider that setting the transmission power to −30 dBm achieves 90% PDR but with 0 dBm only 50% of the packets are received (due to collision). In this case, PC-RPL [10] and active probing [24] choose 0 dBm, and ART [8] will keep changing its transmission power without converging. An example run of TPP is illustrated in Figure 2. TPP considers a window of size *M*, which is managed in a similar way to the PC-RPL transmission power control mechanism. For a fair comparison, TPP initializes the transmission power based on RSSI from the first DIO.

In addition to finding the “optimal” transmission power in a scenario in which no “reliable” link can be found, it is also important to quickly converge. The reinforcement learning formulation allows ACTOR to explore the alternatives efficiently. We show that fast convergence and optimal transmission power control and routing can be achieved, along with stable topology, on resource-constrained COTS devices.

#### 3.3.2. XRPL

XRPL [21] is our second benchmark, which takes a cross-layer approach to jointly adjusting the transmission power and the routes. Their proposal integrates an OF called the Minimum Expected Transmission Power OF (METOF), which basically multiplies ETX with the transmission power. XRPL defines a new Information Element (IE) in the 802.15.4 frame that notifies the receiver of the transmitter’s transmission power. The receiver utilizes this information to calculate its OF and route selection. XRPL is fast to respond to evolving link qualities, as it adapts to the environment upon receiving each packet. On the other hand, XRPL only allows two transmission power settings to simplify the exploration.

## 4. Preliminary Study on the Impact of Transmission Power on the Reliability of Wireless Links

This section analyzes the probability distribution of the delivery of packets. This can be considered a preliminary study that guides the selection of transmission power for ACTOR. This is essential to check the applicability of the UCB algorithms and their variants. Defining tighter upper bounds contributes to minimizing the regret of the UCB algorithm.

In this experiment, two NRF5340 nodes [33] are positioned at specific distances within each other’s line of sight. The two nodes (one as a server and one as a client) are programmed to communicate with different transmission power levels. The client sends 200 batches of packets for each power level to sample the link quality.

In the literature [34], three classes of wireless links are identified, namely (i) connected, (ii) disconnected, and (iii) the links in the transitional region. The former two demonstrate a deterministic behavior; packets either go through or are dropped. However, for the transitional region, an element of randomness is involved.

**Figure 3:**
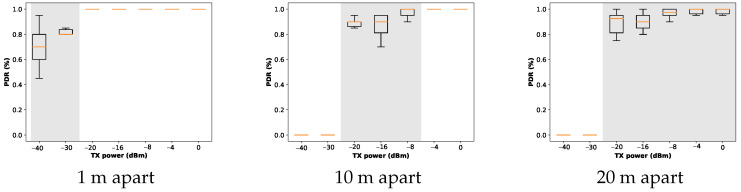
Measured PDR for samples of packets being sent with different transmission power settings at specific distances.

Figure 3 illustrates the probability of delivering the packets being sent over a distance of 1, 10, and 20 m. The box plots show the spread and skewness of the measured data by showing their four quartiles. The transitional region is also determined using a shade of gray. It can be seen that the probabilities for delivery of packets associated with all transmission power settings fall with an increasing distance. For example, when the transmitter and the receiver are only one meter apart, the packets are very likely to be delivered even with the lowest power level, and higher transmission power settings almost surely guarantee the delivery of the packets. On the other hand, at a distance of 20 m, even the highest transmission power cannot guarantee 100% PDR.

Based on the above results, ACTOR blacklists the transmission power settings that lead to disconnected links. Blacklisting speeds up the process of finding the optimal transmission power during the initialization of the protocol, as described in Section 5.2. These results also indicate that, even in simplistic scenarios where only two nodes are involved, a 100% delivery of packets cannot be assumed for higher transmission power settings. This assumption, as far as it is from reality, has been used in the related work to find the best transmission power. In the two-node scenario, this can be due to many reasons, ranging from the shadowing effect to external noise. In a mesh network, the complexity of estimating the link quality tends to increase. These results require us to come up with transmission power control solutions that consider lossy links and the dynamic nature of radio links.

## 5. ACTOR Design

This section describes the design of ACTOR, beginning with an introduction to the building blocks and the design, followed by reviewing each module in detail. Two versions of the algorithm are introduced. First, the basic ACTOR is designed for static environments. The second algorithm, ACTOR-D, differs from ACTOR by implementing the Discounted UCB algorithm while retaining all of the other core features.

### 5.1. The Building Blocks

ACTOR differentiates from the previous works [10,21,24] as follows: Primarily, ACTOR aims to improve the throughput by mitigating the density of the networks and the hidden terminal problem. Consequently, the energy consumption is also reduced due to lower transmission power settings and fewer retransmissions of packets. Second, the selection of the transmission power settings is based on the UCB algorithm. Also, the non-stationary extension (discounted UCB) allows ACTOR to adapt to the dynamism in the links. ACTOR uses lightweight integer arithmetic to simplify the complexities of handling floating point operations. Third, ACTOR updates the ETX as a routing metric to avoid excessive parent changes while the algorithm figures out the best transmission power. Fourth, ACTOR integrates mechanisms to take care of parent–child links while adapting the transmission power so that parents do not neglect the link to children and selfishly reduce their transmission power.

When tuning transmission power based on the reception ratio of the packets, nodes encounter the exploration–exploitation trade-off. That is because, the more often a transmission power is used, the more confident one can get about the reliability of that specific transmission power. The drawback of this exploration is that trying sub-optimal transmission power settings only to evaluate them may cause some packets to be lost. However, a certain level of exploration is inevitable since the agent (node) learns about the environment based on the rewards (acknowledgments) that correspond to the actions (transmission power settings). The ultimate goal of the agent is to find the optimal action with the minimum exploration. Before sending actual packets, the reliability of the links can only be determined by measuring the signal strength and the noise ratio.

Despite a small memory footprint, RPL networks are expected to scale to thousands of nodes. Hence, the protocol limits the number of neighbors in its routing table. In non-storing mode, only the root node keeps track of the downward routes. Both the storing and non-storing modes of RPL favor collection-based traffic. We exploit these design choices in the transmission power control to limit the exploration to a reasonable level. This leads to faster exploration and reduces the size of the routing table, and fewer data packets are sacrificed to evaluate the transmission power settings.

Algorithm 1 summarizes the algorithm used by ACTOR to select the transmission power. Nodes initialize their neighbor table by setting their transmission power to the maximum and adding the neighbor to the table if required. After sending each packet, nodes update their action values. In this case, action values are the ratio of acknowledged packets for each transmission power and parent. Then, they select the action (transmission power and parent) that maximizes the UCB (as defined in Section 5.3). This selection only considers the upward link, but each node must also meet the requirements of its children. In ACTOR, children declare their demands to their preferred parents using a bit in the data packet’s header. Each node uses a transmission power that (i) satisfies its children and (ii) satisfies the UCB requirements.
**Algorithm 1** ACTOR1:Upon receiving a DIO, measure RSSI2:  Initialize the Action Values-Based Equation (5)3:**for** each packet *p* sent through neighbor Nbr **do**4:    **if** Nbr not in the neighbor table AND Received ACK **then**5:        Add Nbr to neighbor table6:        set $N_{t}\left(a\right)$ and ${\bar{X}}_{t}\left(a\right)$ to zero for all transmission power settings7:    **end if**8:    **if** Parent Switch happened **then**9:        Reset the learned Action Values10:    **end if**11:    Calculate UCB index and select transmission power based on based on Equation (8)12:    Select UCB index based on Equation (9)13:    Transmit packet with selected transmission power14:    Increment the counter for transmission power *a*: $N_{t}\left(a\right)=N_{t}\left(a\right)+1$15:    Update $demand_{Nbr}$ from16:    **if** packet sent successfully **then**17:        the Action Values based on Equation (7)18:    **end if**19:    **if** packet sent failed **then**20:        the Action Values based on Equation (7)21:    **end if**22:    Set the transmission power to $\max(UCB,demand_{i})$23:    **if** $A_{t}\neq A_{t-1}$ (if optimal transmission power changes) **then**24:        signals the demand ($A_{t}$) to the parent25:    **end if**26:**end for**

We also help the UCB by filtering the transmission power settings that can be classified as insufficient. If a transmission power has been used at least three times and the action value is zero, we avoid using that action and lower power levels in the future.

It is also worth mentioning that, due to the lack of comprehensive support for floating point operation in the target IoT micro-controllers, we perform the mathematical operations in the integer domain.

### 5.2. Initialization of Action Values

RSSI is not always correlated with PDR due to variations in local link quality [35] and, thus, is not the best candidate for the long-term selection of transmission power. To take external interference and link asymmetry into consideration, ACTOR uses the reception/loss of the packets as the reward. However, for the initialization of the action values, RSSI can be advantageous, as nodes can estimate their distance.

This mechanism requires the DIO packets to be sent with maximum transmission power (0 dBm) or a constant transmission power level noted as $P_{DIO}$. Upon receiving a DIO, nodes measure the loss in the signal strength, ΔP, which is simply the difference between $P_{DIO}$ and the received power, ($P_{RX})$. If we assume the link is symmetric, and the transmission power is configured to *a*, then the received power at the receiver would be a−ΔP. Based on the empirical results from Section 4, we estimate the $\theta_{-}$ and $\theta^{+}$, which are the minimum and maximum RSSI measurements that put the link in the transitional region.

ACTOR initializes the Action Values in three classes, as in Equation (5). Simply put, $X_{0}\left(a\right)$ is the estimated value of the PDR of the link if the transmission power is set to *a*. If a certain transmission power puts the link in the transitional region, the PDR is set using a linear function of the power loss and the difference between $\theta^{+}$ and $\theta_{-}$ [34]. This initialization is performed quickly, but it needs to be augmented later with the probing mechanism since it cannot capture noise or link asymmetry.

(5) $$X_{0}\left(a\right)=\left\{\begin{array}{cc} 1, & \mathrm{if}\;\theta^{+}<a-\Delta P\\ \frac{a-\Delta P}{\theta^{+}-\theta_{-}}, & \mathrm{if}\;\theta_{-}<a-\Delta P<\theta^{+}\\ 0, & \mathrm{if}\;a-\Delta P<\theta_{-} \end{array}\right.$$

### 5.3. Upper Confidence Bound (ACTOR)

The optimal regret achieved through the UCB algorithm is the primary driving force of ACTOR. The UCB algorithm uses the upper bounds proposed in the previous section to handle the exploration–exploitation trade-off.

The UCB algorithm measures the confidence in the knowledge of the rewards of each arm in which the term arm corresponds to the transmission power settings. The agent keeps track of the number of times each node has been selected, based on which the UCB index is calculated. The UCB index expresses the estimated upper bound for the rewards that an arm can achieve. At each iteration, the agent chooses the action with the maximum UCB index calculated as in Equation (7) [36].

(6) $${\mathrm{UCB}}_{a}\left(t\right)={\bar{X}}_{t}\left(a\right)+\sqrt{\frac{2\sigma^{2}\log\left(t\right)}{N_{t}\left(a\right)}}$$

(7) $$Action\left(t\right)=\arg\max_{a\in A}\left[{\mathrm{UCB}}_{a}\left(t-1\right)\right]$$

The first term, ${\bar{X}}_{t}\left(a\right)$, denotes the empirical mean of the rewards associated with action *a* by time *t*. Equation (8) presents a recursive definition of the empirical mean of rewards. The reason we use a recursive method, rather than a simple $\frac{1}{n}\sum_{t}X\left(t\right)$, is to reduce the memory footprint on the resource-constrained device. *r* represents the most recent reward. $N_{t}\left(a\right)$ is the number of times action *a* has been chosen until time *t*. For the calculation of σ, we use Lemma 1.

(8) $${\bar{X}}_{t}\left(a\right)=\frac{{\bar{X}}_{t-1}\left(a\right)\cdot N_{t}\left(a\right)+r}{N_{t}\left(a\right)+1}$$

The second term is known as the exploration bonus or confidence width, and it denotes the degree of confidence in the expected reward, and it decreases with the number of times that arm is pulled. UCB shows more of a tendency to explore arms if they are either (a) promising, because ${\bar{X}}_{t}\left(a\right)$ is large, or (b) not well explored due to a large confidence width.

Blacklisting of the futile transmission power settings (based on RSSI) is performed during the initialization. In the example illustrated in Figure 1b, for the lowest three transmission power settings, the link is put in a disconnected region. Hence, ACTOR sets the PDR to 0 and virtually increases $N_{a}\left(t\right)$. This will reduce the tendency of the exploration strategy to select those power levels.

### 5.4. UCB for Non-Stationary Bandits (ACTOR-D)

The basic version of UCB assumes that the distribution of rewards for each arm (transmission power) is stationary in time. However, that is rarely the case, and these distributions are subject to severe change due to evolving environments and mobility [37]. To handle non-stationary rewards, Garivier [18] proposes a variant of UCB called the Discounted UCB.

The Discounted UCB alters the calculation of the first term in the UCB index by applying an adjustable discount factor (λ) to the older observations, giving more importance to the new ones. Equation (9) determines how the discounted UCB calculates rewards.

(9) $${\bar{X}}_{t}\left(a\right)=\frac{{\bar{X}}_{t-1}\left(a\right)\cdot \left(RewardScale-\lambda \right)+r\cdot \lambda }{RewardScale}$$

RewardScale is the maximum possible value for the rewards (100). For tuning λ, a simple choice is to resemble the weight that Contiki-NG RPL assigns to the new packet’s ETX in the routing [38]. Since there is no need to keep track of previous rewards, the Discounted UCB occupies a smaller space in the memory. Given the memory limitations, we chose the Discounted UCB algorithm to be integrated with ACTOR.

The use of bandit models in embedded systems has been minimal due to practical limitations such as missing floating point operations and a small memory capacity. To tackle this challenge, fixed-point arithmetic has been proposed in the literature. Krentz et al. [36] propose methods to efficiently calculate two variants of the UCB algorithm (namely the Discounted UCB and the Sliding Window UCB) for the channel selection problem in IEEE 802.15.4. Through Monte Carlo simulations, they also showed that the accuracy loss in their method is negligible.

ACTOR requires efficient algorithms to determine the natural logarithm and square root. The natural logarithm is easily convertible to base-2 since $\log_{e}\left(x\right)=\log_{2}\left(x\right)/\log_{2}\left(e\right)$. Then, the binary logarithms can be calculated via repeated squaring and dividing [39].

For the implementation of the square root, efficient algorithms exist. The simplest algorithm consists of a linear search that starts checking all the second power of all integers starting from 2 and returns the maximum integer that has a square that is smaller than the input. A more efficient solution improves this search to be binary. All of these methods work fine to determine the UCB.

### 5.5. Topology Control

Providing connectivity for the farther nodes is not a concern when selecting the transmission power in a single-hop setting, but ACTOR is designed for multi-hop networks. A selfish parent could reduce the transmission power and increase its own battery life, leaving its children to find other parents. Another potential problem is that the abrupt changes in the topology may trigger global/local repair mechanisms that incur an overhead of the control packets when the nodes are changing their transmission power.

**Figure 4:**
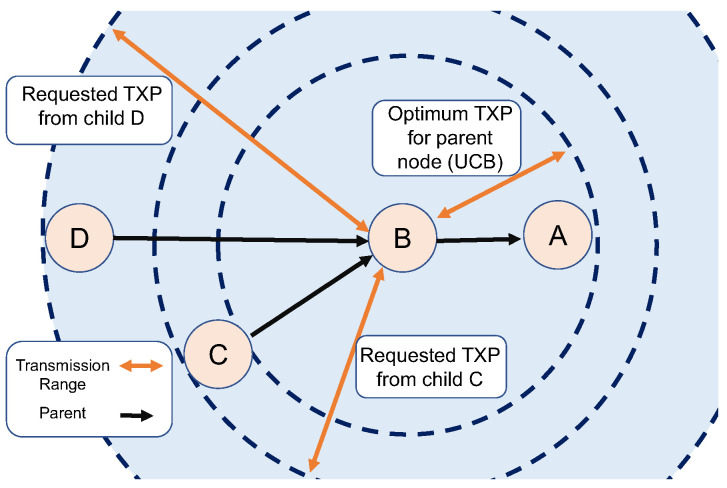
In ACTOR, the parent node (node B) commits to the requirements of children (nodes C and D) to keep the topology stable.

ACTOR’s topology control mechanism tries to avert excessive changes in the RPL’s DODAG. When children acquire a level of certainty in their desired transmission power, they demand the parent keep their transmission power at least as high. In other words, nodes send a packet to their parent asking for a minimum transmission power. As illustrated in Figure 4, the parent node can choose a low transmission power for its own data packets, but it commits to higher levels to support its children. Topology control not only avoids the RPL’s repair mechanism but also keeps the children connected.

### 5.6. Routing Metric

ETX is the most commonly used routing metric for RPL. We updated ETX to account for the transmission power control mechanism. Calculating ETX in the open implementation of Contiki consists of applying an *Exponentially Weighted Moving Average* (EWMA) to the ETX, as in Equation (10). The factor α determines the weight based on which new observations are preferred to the old ones.

(10) $${\bar{ETX}}_{t}\left(p\right)=\frac{{\bar{ETX}}_{t-1}\left(p\right)\cdot \left(Scale-\alpha \right)+PacketETX\cdot \alpha }{Scale}$$

ACTOR’s calculation of ETX must consider that the transmission power is changing. For example, if a node temporarily tries to evaluate a link with a low transmission power, the ETX associated with this transmission power should not be advertised to the farther nodes. Hence, for each neighbor, ACTOR maintains a table (rather than a single value) for the ETX associated with each transmission power, according to Equation (11).

(11) $$ETX_{t}\left(p,a\right)=\frac{ETX_{t-1}\left(p,a\right)\cdot \left(Scale-\alpha \right)+PacketETX\cdot \alpha }{Scale}$$

When the trickle algorithm triggers the transmission of a DIO, the ETX value that is advertised is associated with the transmission power that maximizes the ETX index at that point in time.

$$ETX_{t}\left(p\right)=\max_{A_{t}\in A}ETX_{t}\left(p,A_{t}\right)$$

## 6. Evaluation

In this section, we evaluate the performance of ACTOR and ACTOR-D, and we compare them with standard RPL (with the maximum transmission power) and our two selected benchmarks, TPP and XRPL. The evaluations are based on both simulations and a physical testbed that consists of NRF5340 boards. Due to compatibility issues with the hardware platform in our testbed (NRF5340), XRPL was evaluated only in the simulations. We first took a look at the setup for the simulations and the physical testbed and then analyzed the results obtained using each of them.

We used a single-channel CSMA for the communication between nodes to show that the proposed method succeeds in reducing channel contention. RPL’s objective function is set to *Minimum Rank with Hysteresis Objective Function* (MRHOF) with ETX as the routing metric. We repeated the experiments during working hours and after working hours to confirm the results, with each run lasting 10 min (both in experiments and the simulation), which gave the protocol enough time to converge and be fairly compared. To support reproducible results and measurable confidence, we benefited from a recent framework called TriScale [40]. At least five independent runs are required to estimate the mean of a measured metric with a probability of 95 percent. We show the 95% confidence interval (a minimum and a maximum of five runs) in the results using error bars.

### 6.1. Simulation Setup

The simulations were conducted using the built-in simulator of Contiki, Cooja, which comes with plenty of options to model the radio models, including the *Unit Disk Graph Model* (UDGM) and the *Logistic Loss Model* (LLM). The UDGM model plots two disks around each node, representing the connectivity range and the interference area. LLM provides a more realistic model, as it uses the logistic function to calculate the reception probability of packets based on RSSI. The simulated boards are Sky Motes with TI’s MSP430 F1611 controller with 10 kB of RAM and 48 kB of flash. They are equipped with a cc2420 radio that supports eight different settings for transmission power. This choice of sky motes was made to demonstrate the possibility of running the algorithm on legacy COTS since, in the experiments, we used a rather modern board. The current consumption associated with all the transmission power settings for sky motes is detailed in Table 1. For the NRF motes that are used in the experiments, the possible transmission powers are shown in Table 2.

We considered 7 different scenarios, as detailed in Table 3. The parameters in these scenarios were chosen deliberately to exemplify challenging conditions for routing and transmission power control.

**Figure 5:**
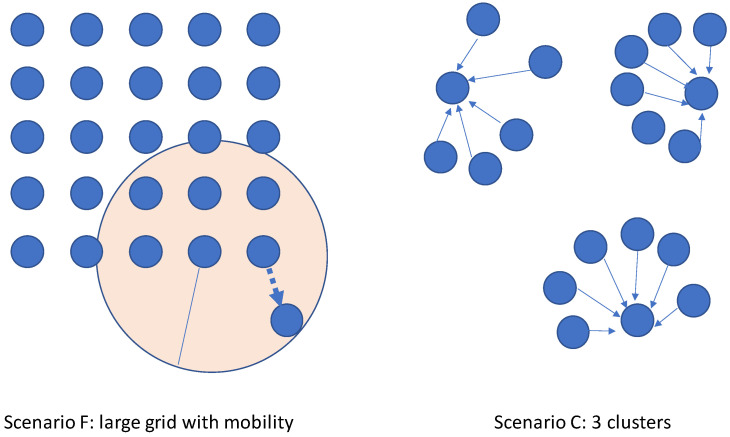
Simulation topology for scenarios F and C.

- Scenario A represents a small-scale network with nodes positioned at distances where there is no benefit in reducing the transmission power. This scenario portrays a sparse deployment that is challenging for ACTOR. In such a deployment, ACTOR is expected to converge on high transmission power settings, and trying lower transmission power will only waste resources.
- In scenario B, the parameters were chosen to exhibit density. Forty nodes were positioned randomly in a 100×100 square. If the RPL nodes transmitted at their maximum power (0 dBm) with a 50 m range, then most of the nodes would have a high number of neighbors (at least 20).
- Scenario C (illustrated in Figure 5) resembles a multi-cluster topology, and it is based on the multi-instance feature of RPL. If the nodes in a cluster manage their transmission power properly, the clusters will not interfere with each other. The hop distance of the nodes is less than in the previous scenario. With fewer children, parent nodes have more freedom to tune their transmission power.
- In scenario D, a high load of traffic is applied to a medium-dense network.
- Scenario E presents the same 5 × 5 grid with a lower link quality, and it uses the more realistic LLM link model.
- Scenario F includes a 5 × 5 grid and one mobile node. The mobility pattern is simple, and it can be dealt with using only the transmission power control. This topology is illustrated in Figure 5.
- Scenario G tests the scalability of the system in a topology with 100 randomly placed nodes.

### 6.2. Experimental Setup

Conventional simulators and their radio models fail to accurately model physical characteristics such as the capture effect or the distribution of the noise since they are too simplistic and usually overlook some details. For radios with narrow band modulation, it is not only the receiver threshold and non-ideal antennas that cause the transitional region. Multi-path fading plays a major role [34] in the connectivity of these radios, and transmission power control is a decisive parameter in managing the shadowing effect. Most simulators for low-power radios fall short of modeling the shadowing effect by considering a log-normal shadowing model. Real-world experiments are undeniably more accurate in this matter.

In order to obtain more accurate evaluations, we assembled a physical testbed containing 40 NRF5340 boards. The experiments were conducted using the modern NRF5340 development boards introduced by Nordic Semiconductor [33] featuring two Arm Cortex-M33 processors. The first processor, referred to as the network core, comes with 256 kB of flash and 64 kB of RAM, and it runs at a low power mode, while the application core provides a considerably higher computation capacity. To optimize the power consumption, this application core gives up control to the network core upon initialization and remains inactive thereafter.

According to the datasheets, the NRF5340 affords the flexibility to configure 24 distinct settings for transmission power, offering a finer granularity of control over the transmission power in comparison to the eight levels available in sky motes [41]. In an effort to maintain a fair utilization of system memory, we opted to restrict the NRF5340 to employ eight transmission power settings. As shown in Table 2, NRF5340 consumes less energy compared to sky motes, owing to its more recent radio.

**Figure 6:**
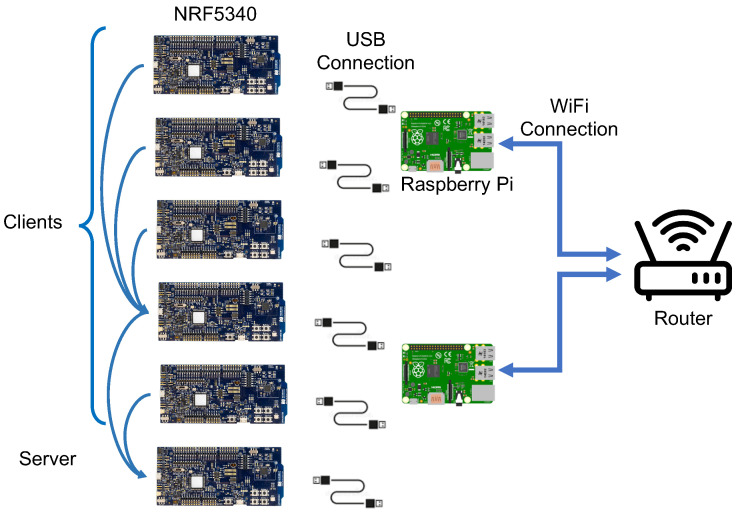
The configuration for programming the nodes and gathering measurements from the testbed involved utilizing a Raspberry Pi model 4B, which interfaced with the NRF nodes via serial connections, supplemented with Wi-Fi connectivity for broader functionality.

Part of the experimental setup is illustrated in Figure 6. Ten Raspberry Pis (only two are shown) are powering up and controlling the NRF5340 boards. The Raspberry Pis are then managed through a *Secure Shell* (SSH) protocol over a Wi-Fi access point configured to operate on a specific channel so that it does not interfere with the operation of NRF boards.

**Figure 7:**
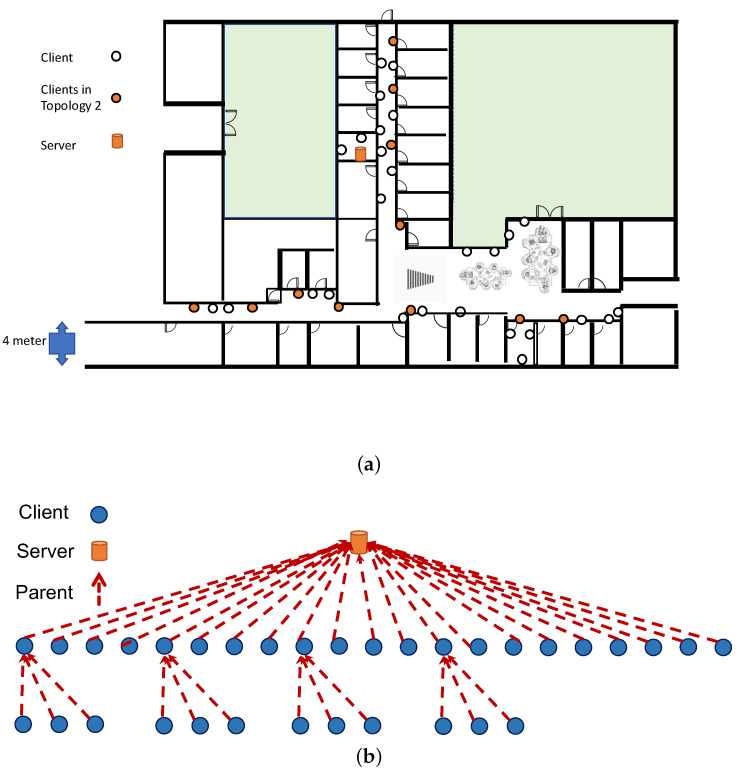
The NRF5340 nodes are placed in the corridors of the Engineering School at Mälardalen University (**a**). The 12-node experiment only used the orange nodes, while in the 40-node experiment, all the nodes were used. The most recurring topology that the network converges to is a tree with a maximum distance of two hops (**b**).

The testbed was located in the corridors and rooms of the IDT school of the Mälardalen University, as illustrated in Figure 7a. The experiments were performed using two settings with (i) orange nodes establishing a sparse topology for a 12-node scenario and (ii) utilizing all 40 nodes for a dense deployment. The running topology (for both ACTOR and default RPL) is dynamic, and it depends on time and the randomness caused due to the trickle algorithm that triggers the dissemination of DIO packets. The most recurring topology consists of almost half of the nodes being connected in a single hop, while the rest are being connected with two hop links (as demonstrated in Figure 7b. ACTOR-D and standard RPL ended up having very a similar topology but with a much lower rate of parent switching. Analyzing the normal Wi-Fi traffic in the office environment showed that channel 24 in IEEE 802.15.4 was the least disrupted. We fixed the communication of our testbed on this channel to minimize Wi-Fi interference.

### 6.3. Simulation Results

**Figure 8:**
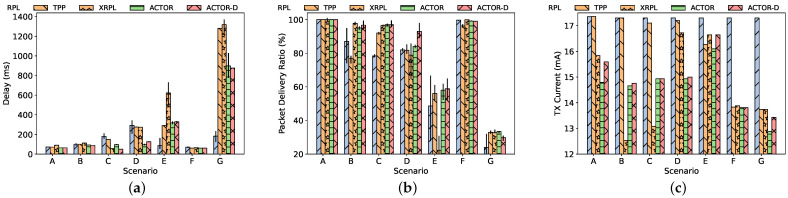
Simulation results considering seven different scenarios showing: (**a**) End-to-end delay, (**b**) Packet Delivery Ratio, and (**c**) the current used for radio transmission.

Figure 8 summarizes the results of the conducted simulations in terms of PDR, the end-to-end delay, and the current used for transmission. Figure 8b plots the average PDR and shows that ACTOR and ACTOR-D achieve a lower packet loss compared to RPL and the benchmarks in dense (B), multi-cluster (C), high-rate (D), lossy (E), mobile (F), and 100-node (G) scenarios. This is mainly due to spatial reuse in ACTOR that overcomes the congestion in the wireless medium. In a dense network, ACTOR exhibits better resilience against heavy traffic loads compared to TPP and XRPL. In scenario A, ACTOR leads to negligible loss (attributable to exploration), indicating that reducing the transmission power in a sparse network is not as beneficial as in a dense network. Under ACTOR, CSMA nodes happen to have fewer backoffs and failed CCAs. This leads to a significant improvement in the delay for ACTOR in scenarios C, D, E, and G which is demonstrated in Figure 8a. Like PDR, ACTOR outperforms RPL, TPP, and XRPL in terms of end-to-end delay in the dense scenarios, confirming that ACTOR reduces channel contention. In order to test the scalability of the system, we included a scenario with 100 randomly positioned nodes. As you can see, in scenario G, ACTOR improves the delay and current consumption (Figure 8c) while providing the same level of PDR. Please note that, in scenarios E and G, RPL provides a very small delay, which is irrelevant since most of the packets are dropped by RPL. In ACTOR, all the nodes send a packet to their parents to indicate the minimum required transmission power to agree on a transmission power that does not jeopardize the routing protocol. When normal data transmission is challenged due to intensive collisions, the overhead caused by the negotiation can be excessive. Specifically, in scenario G, ACTOR nodes transmitted 12 thousand extra packets for transmission power negotiations with all 100 of the nodes during the 10 min, while the transmitted data packets summed up to almost 30 thousands. Despite this overhead, the optimal selection of transmission power of ACTOR led to good results in such large scale.

We also measured the current consumption using Energest (https://docs.contiki-ng.org/en/develop/doc/programming/Energest.html accessed on 3 January 2024), which is a software-based energy estimation module. Figure 8c depicts the average current consumption for all of the nodes. ACTOR consistently (even in a sparse scenario) achieves lower energy consumption due to its regret-optimal transmission power selection. Reducing the transmission power not only reduces the per-transmission energy but also leads to fewer retransmission of packets, further improving the energy consumption. ACTOR nodes report (i) less time spent with their radio in transmission mode and (ii) a lower transmission power. In the dense and multi-cluster topology, the benefits of employing ACTOR are even more palpable.

In the sparse topology (A), the nodes cannot meaningfully reduce their transmission power. Hence, ACTOR cannot demonstrate its advantages in PDR, and there is only a small gain in terms of energy consumption. Nevertheless, ACTOR may sacrifice a few data packets to explore lower transmission power settings that are not helpful in a sparse setting, and this leads to lower PDR. ACTOR tries to minimize this so-called *exploration loss* to a negligible level via its blacklisting mechanism and the UCB exploration strategy. As we can see in the transition from scenarios A to G as the topology becomes dense, the exploration loss becomes more important. In most of the scenarios, ACTOR outperforms XRPL. The advantages of optimal exploration (UCB) are more evident in the scenario with unreliable links (E), as XRPL shows a lower PDR and higher delay without being able to reduce its transmission power. In the scenario with mobility (F), we see that ACTOR manages to keep the connectivity better than TPP and almost at the same level as RPL (which performs well due to its high transmission power). However, it is worth noting that sustaining connectivity for mobile nodes is even challenging for ACTOR, as the previously established rewards have become irrelevant.

### 6.4. Experimental Results

**Figure 9:**
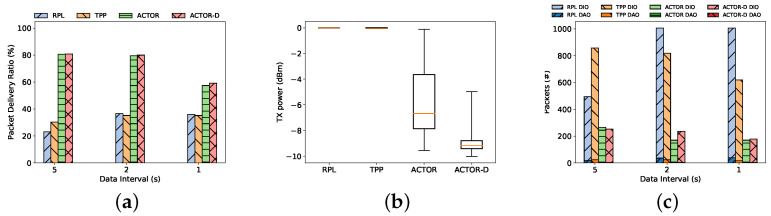
Experiment results for the 40-node scenario show ACTOR outperforming the benchmarks at different data intervals showing: (**a**) Packet Delivery Ratio, (**b**) Average transmission power, and (**c**) Routing overhead.

Using the testbed that we described previously, we compared the performance of two versions of ACTOR with the baseline, RPL, and the benchmark, TPP. Figure 9 shows the advantage of using ACTOR in terms of PDR, transmission power, and routing overhead as the traffic increases.

Figure 9a plots the aggregate packet delivery ratio of ACTOR, ACTOR-D, TPP, and standard RPL with different data intervals. The results suggest that the two versions of ACTOR provide better throughput (PDR) than TPP and RPL. When a smaller volume of traffic is applied to the network (5-s interval), ACTOR beats both RPL and TPP by almost 40 percent. In other words, ACTOR reduces the packet loss rate by four times compared to the standard.

The advantages of using ACTOR in this scenario are better shown in the experiments than in the simulations. This stems from the fact that, in a realistic environment, none of the links are ideal. As we saw in Section 4, links behave randomly in the transitional region. In this situation, benchmarks cannot find a transmission power that transmits a number of consecutive packets successfully and select the maximum transmission power. UCB is able to find the best transmission power in those cases, but the benchmarks fails to adapt their simplistic transmission power control. The other reason is the local repair mechanism in the RPL protocol. Once nodes detect an abrupt change in the measured routing metric (ETX), they initiate the local repair mechanism of RPL, which consists of disconnecting from the parent and sending a DODAG Informational Solicitation (DIS) asking for a new parent. This can be seen in Figure 9c, which shows that the local repair mechanism causes more control packets (both DIO and DAO) for RPL and TPP compared to ACTOR.

ACTOR-D slightly outperforms ACTOR in the 40-node experiment, thanks to the discounting mechanism. Nodes that keep changing their transmission power impair the wireless links belonging to other nodes. Hence, when nodes are not moving or when there is a dynamic source of interference, this may be the source of dynamism in the quality of the links, and ACTOR-D is tailored to handle this.

Figure 9b that ACTOR and ACTOR-D successfully reduce the transmission power while providing better connectivity. For RPL, the transmission power is always at 0 dBm. The quartile chart shows that TPP nodes barely try lowering their transmission power and converge on the maximum. In addition, ACTOR improves the average number of retransmissions for all of the nodes in the network. This metric depends on (i) the number of hops and (ii) single-hop contention. Since the number of hops has not changed, a higher number of retransmissions indicates more hidden terminals for the medium in the MAC layer. We can see that ACTOR succeeds in reducing the hidden terminals.

In addition to the transmission power, another popular best practice to reduce energy consumption is to reduce idle listening by allowing the node to put the radio in sleep mode [42]. Although the results presented here considered CSMA, ACTOR is expected to be beneficial for other MAC protocols as well. For example, TSCH networks take advantage of diversity in both the frequency and time domains. TSCH nodes agree on a schedule to use channels in time (time is divided into timeslots). These schedules contain shared timeslots for broadcasts, reserved timeslots for unicast communications between pairs, and also idle timeslots for the radio to sleep. The scheduler needs to take the interfering nodes into account when assigning reserved slots. Optimizing the transmission power leads to fewer interfering nodes and fewer constraints for the scheduler. However, transmission power control in a duty cycling network is considered out of the scope of this work.

We also performed the experiments in a scenario that consists of 11 clients and 1 server providing a sparse topology, as illustrated using orange circles in Figure 7a. The measurements are presented in Table 4. Another significant aspect is keeping the convergence time of the routing protocol short. The number of parent switches can be an indication of the convergence time of the routing protocol. The results also showed that ACTOR did not increase the hops distance of the nodes, reduced the transmission power, and significantly reduced the number of parent switches. Overall, the experimental results are in line with the simulations, as they both show the advantages of tuning the transmission power when wireless links are unreliable and network is dense.

## 7. Conclusions

This paper has proposed ACTOR, an extension to RPL that adapts the transmission power using reinforcement learning. The routing and transmission power control mutually improve each other. The information derived using the routing protocol reduces the number of transmission power settings that must be evaluated. Transmission power control also helps RPL keep the topology stable by mitigating the interference among the nodes. We have provided compelling evidence, including simulation and experimental results indicating that the proposed method improves the performance of multi-hop networks in different scenarios, such as dense networks and a high traffic load.

The results are indicative of a positive correlation between the success of the exploration strategy to optimize the transmission power and networking performance. The results vary, depending on the scenarios, but generally for dense networks, the experiments showed a 20–40% increase compared to the benchmark in PDR, as well as the achievement of up to 10 dBm lower transmission power. This enhancement is accompanied by a more stable topology and lower routing overhead, which are attributable to the better management of channel contention. The main catalyst behind the results is the intelligent UCB and discounted UCB algorithm, which explores the transmission power settings with an intelligent mechanism.

As future work, we plan to integrate ACTOR with solutions for the mobility of the nodes under which RPL exhibits poor performance. The support for a dynamic environment can be extended to mobility, but it requires the tailoring of the exploration strategy and integration with the existing mobility management solutions.

## Figures and Tables

**Table 1:** Sky mote’s current consumption for different transmission power settings (used in simulations).

Transmission Power (dBm)	Current (mA)
0	17.4
−1	16.5
−3	15.2
−5	13.9
−7	12.5
−10	11.2
−15	9.9
−25	8.5

**Table 2:** NRF5340’s current consumption for different transmission power configurations (used in the experimental testbed).

Transmission Power (dBm)	Current (mA)
+3	5.1
0	3.4
−4	2.7
−8	2.2
−12	2.0
−16	1.8
−20	1.7
−40	1.5

**Table 3:** Simulation scenarios and parameters.

Scenario	Topology	Clients/Servers	Traffic Load (pkt/min/node)	Explanation
A	Random	10/1	6	Upward traffic + sparse
B	Random	40 /1	6	Upward traffic + dense
C	Three-cluster	30/3	6	(Up + Down)−ward traffic
D	Random mid-density	20/1	60	Upward traffic + congestion
E	Grid 5×5 unreliable links	25/1	6	RX Ratio = 0.7, Radio = LLM
F	Grid 5×5 mobile node	25/1	6	Mobile nodes = 1
G	Random	100/1	30	In a 200 × 200 m area

**Table 4:** Measurements for the 12-node scenario.

Protocol	RPL	TPP	ACTOR	ACTOR-D
PDR	59.09%	73.4 %	82.5%	79.84%
Transmission power (dBm)	0	−6.5	−7.39	−7.95
Routing overhead (pkt/node)	12	7	6	7
No. of retransmissions	17.3	14.8	12.77	13.58
No. of parent switch	1.8	1.45	1.18	1.63

## Data Availability

Data are contained within the article.

## Appendix A. Proof of Lemmas Used for the Upper Confidence Bound

**Lemma** **A1** (Hoeffding’s Lemma)**.** *If a≤X≤b with probability 1, then X is a $\frac{{\left(b-a\right)}^{2}}{4}$-subgaussian random variable.*

**Proof.** In the first step, we prove the following for any random variable, *X*:

$$\begin{array}{c} Var\left(X\right)=\underset{t}{\inf}\left[{\left(X-t\right)}^{2}\right]\\ =Var(X-\frac{{\left(b-a\right)}^{2}}{2})\\ \leq \frac{{\left(b-a\right)}^{2}}{4} \end{array}$$

Now, let *P* denote the distribution of *X* and define $\rho \left(\lambda \right)=\log E_{p}\left[e^{\lambda X}\right]$. Let $Q_{\lambda }$ be the distribution of *X*, defined as follows:

$$dQ_{\lambda }\left(x\right)=\frac{e^{\lambda x}}{E_{P}\left[e^{\lambda X}\right]}dP_{X}$$

$$\rho^{\prime }\left(\lambda \right)=\frac{E_{P}\left[Xe^{\lambda X}\right]}{E_{P}\left[e^{\lambda X}\right]}=\int x\frac{e^{\lambda X}}{E_{P}\left[e^{\lambda X}\right]}dP\left(x\right)=E_{Q}\left[X\right]$$

$$\begin{array}{c} \rho^{\prime \prime }\left(\lambda \right)=\frac{E_{P}\left[X^{2}e^{\lambda X}\right]}{E_{P}\left[e^{\lambda X}\right]}-\frac{E_{P}{\left[Xe^{\lambda X}\right]}^{2}}{E_{P}{\left[e^{\lambda X}\right]}^{2}}=\\ E_{Q}\left[X^{2}\right]-E_{Q}{\left[X\right]}^{2}=Var_{Q}\left(X\right) \end{array}$$

From Step 1, we have $\rho^{\prime \prime }\left(\lambda \right)=Var_{Q}\left(X\right)\leq \frac{{\left(b-a\right)}^{2}}{4}$. According to Taylor’s theorem, there exists some $\tilde{\lambda }\in \left[0,\lambda \right]$ such that

$$\rho \left(\lambda \right)=\rho \left(0\right)+\rho^{\prime }\left(0\right)\lambda +1/2\rho^{\prime \prime }\left(0\right)+\rho^{\prime \prime }\left(\tilde{\lambda }\right)\lambda^{2}$$

Given the convexity of $e^{X}$, we can exponentiate both sides, and that finalizes the proof. □
